# Buffalo Medical and Surgical Association

**Published:** 1885-07

**Authors:** 


### Society Reports.

### Buffalo Medical and Surgical Association.

*Meeting of May 5, 1885.*

The President, Dr. Charles G. Stockton, in the Chair; Dr. Frank H. Potter, Secretary.

The names of Drs. H. Bauer, S. T. Howell and F. R. Campbell were presented for membership, and upon separate ballots they were declared duly elected.

Dr. Stockton then delivered his inaugural address, in which he called attention to the position the Society had occupied in the profession in former times, and mentioned some of the illustrated names connected with it. He had two suggestions to make for the work for the coming year: 1st. That the papers presented to the Society should receive, and were entitled to receive, the freest criticism. It was only in this way that the subjects considered could be thoroughly brought before the Society, and the differences of opinion in the profession made manifest. He hoped the discussions during his term as President would be made one of the interesting features of the meetings. 2d. He thought that in this matter of criticism too much deference was paid to authority. Each man's opinion should be presented without fear, even if they differed from those held by others whose teachings are received with respect by the profession. As an illustration, he instanced the opinion of Prof. Austin Flint upon the question of diet. The advice given by the latter to eat as much as you liked and whenever you were hungry, he considered to be at variance with sound physiological principles. Only one step more was needed, and we would be told that mastication was unnecessary, and that we could get along just as well by "bolting" our food, like the anaconda.

Dr. James W. Putnam then read the paper of the evening, entitled the "Causes and Treatment of Lithæmia." This paper will appear in a future number of the Buffalo Medical and Surgical Journal, and an abstract is, therefore, omitted.

In the discussion—

Dr. F. W. Bartlett reported a case in which the symptoms had been severe, but which had yielded to treatment similar to that advised by Dr. Putnam. He called attention to the fact that the nails often presented transverse, instead of longitudinal, lines in patients suffering from lithæmia, and said that Bright's disease and some other diseases, such as endocarditis, might often be primarily cases of lithæmia.

Dr. M. D. Mann had had some personal acquaintance with the disease in question. He believed heredity to be a very important factor in the disease. Some members of a family might suffer from gout, others from rheumatism, and still others from lithæmia, as it had been described by Dr. Putnam. He had also observed that women at the menopause frequently suffered from this disease. Among the symptons of which they complained at this time, he specially mentioned great irritability of the bladder and a watery leucorrhœa associated with an elytritis of a low grade. These and their other symptoms might often be made to disappear by careful attention to the diet and exercise. As to treatment, he preferred a meat to a vegetable diet. He thought Dr. Putnam had not sufficiently considered the important part the starches and the sugars played in the production of lithæmia. It was important to avoid the use of them altogether, if possible, in these cases. It was also necessary for the patient to abstain from alcohol, beer, and the sweet wines, as they were potent factors for evil. Thorough mastication was likewise an important point in treatment.

Dr. Cronyn thought the question of heredity had some bearing on the difference in the lines of treatment advocated by Dr. Mann and Dr. Putnam. He called attention to an order of the Roman Catholic Church—the Trappists—whose members never suffered with gout. They lived exclusively upon a vegetable diet and drank no wine. They generally attained to a very old age.

Dr. Hinkel considered lithæmia a not infrequent cause of recurrent amygdalitis. Catarrhal conditions of the mucous membranes of the air-passages were also produced by it. He believed in the line of treatment advocated by Dr. Mann.

Dr. W. S. Tremaine had suffered from the disease, and had tried various sorts of treatment with varying degrees of success. He believed that, on the whole, the avoidance of the starches and sugars would give the best results. Mastication he considered of the greatest importance. This was apt to be neglected, and it was necessary to instruct the patient particularly on this point.

Dr. Wetmore had also suffered from the disease, and added his testimony to that of the others concerning its symptoms and treatment. In his case, one of the earliest symptoms was a sore throat. He believed there was reason in both methods of treatment, and that the cases should be carefully classified before the treatment was decided upon. A peculiarity of some cases was in the urine remaining normal, not showing a deposit, as was observed in the majority of cases.

Dr. Tremaine confirmed this observation about the urine. He said its acidity was not increased, although the patient presented many of the symptoms due to the lithæmic condition.

Dr. Putnam said he always prohibited the sugars and alcoholic liquors, as well as all fried meats, pork, bacon, etc. He believed that the difference in the results was due to the difference in the cases.

The quantity of food was as important as its quality. Too large an amount favored the production of lithæmia, from the inability of the system to assimilate it. The tendency of many cases of gonorrhœa to become gleet was due to the lithæmic condition, and relief was obtained by the alkaline treatment.

The prevailing diseases reported were measles, scarlet fever, pneumonia and inflammatory rheumatism.

The President appointed Drs. F. W. Hinkel, J. W. Putnam and E. H. Long as the standing Financial Committee for the ensuing year.

The Society then adjourned.

